# Hepatoprotective effect of nanoniosome loaded *Myristica fragrans* phenolic compounds in mice‐induced hepatotoxicity

**DOI:** 10.1111/jcmm.17581

**Published:** 2022-10-12

**Authors:** Mahsa Rastegar Moghaddam Poorbagher, Ehsan Karimi, Ehsan Oskoueian

**Affiliations:** ^1^ Department of Biology, Mashhad Branch Islamic Azad University Mashhad Iran; ^2^ Department of Research and Development, Arka Industrial Cluster Mashhad Iran

**Keywords:** antioxidant, drug delivery, encapsulation, L‐asparaginase, liver injury, nanotechnology

## Abstract

In this study, nanoniosome‐loaded *Myristica fragrans*' (MF) phenolic compounds (NLMP) were synthesized and characterized for their physical properties, and hepatoprotective effects on mice with liver toxicity induced by L‐asparaginase (LA) injection. According to the results, NLMP has a spherical shape with a 263 nm diameter, a zeta potential of −26.55 mV and a polydispersity index (PDI) of 0.192. The weight and feed intake of mice induced with hepatotoxicity were significantly (*p* ≤ 0.05) increased after they were treated with NLMP (2.5 mg/kg body weight of mice). In addition, the blood levels of triglyceride (TG), cholesterol (Chol), liver enzymes (aspartate aminotransferase (AST), alanine transaminase (ALT), alkaline phosphatase (ALP)) and total bilirubin were significantly (*p* ≤ 0.05) decreased. A significant increase (*p* ≤ 0.05) in the blood levels of the antioxidant defence system (glutathione peroxidase (GPX), superoxide dismutase (SOD) and catalase (CAT)) were also reported after NLMP treatment. NLMP was also led to a significant decrease (*p* ≤ 0.05) in inflammatory‐related gene expression of inducible nitric oxide synthase (iNOS) and Interferon‐gamma (IFN‐γ) in the liver, as well as a meaningful (*p* ≤ 0.05) increase in the expression of SOD as an antioxidant status biomarker. Consequently, the NLMP is recommended as a potential dietary supplement to alleviate the symptoms of LA‐induced hepatotoxicity.

## 1 INTRODUCTION

The liver is the most significant organ for the detoxification of toxic substances in the human body. As a biological barrier, it can remove different exogenous substances through phagocytosis.^1^ Drug‐induced liver injury is an extended term that refers to any hepatic injury which results from a prescribed medication.^2^ LA is an antineoplastic agent, which is utilized as a therapy for acute lymphoblastic leukaemia (ALL). Asparagine is an essential amino acid for leukaemia cells' growth, but they lack asparagine synthase and rely upon the host's asparagine supply for protein production. LA act as an anti‐ALL by catalysing Asparagine degradation into aspartate and ammonia and decreasing the glutamine pool.^3^ Amino acid reduction results in depletion in protein synthesis, causing mitochondrial dysfunction, metabolic stress and ROS generation in leukaemia cells, mediating their death. However, this reduced protein synthesis leads to impaired mitochondria β‐oxidation and unoxidized fatty acid accumulation in the hepatic parenchyma. This can lead to hepatocyte necrosis, which is not very notable, though steatosis is usually seen in 50%–90% of occasions.^4^ Mild elevation of transaminases and bilirubin to severe hepatic failure, multi‐organ dysfunction and death are ranges of LA‐induced hepatotoxicity.^5^ Stress‐related genes such as catalase (CAT) and glutathione S‐transferase (GST) and liver metabolism FADS2 gene were shown to be increased after treatment with LA in zebrafish.^6^ Anti‐asparaginase IgG antibodies were also developed and caused hypersensitivity by mice that received commercial E. coli LA.^7^ In one of the previous studies, a decrease in serum albumin and increases in ALT and fatty liver were observed in hepatic imaging of patients after LA utilization.^8^, ^9^ In addition, LA was shown to reduce fibrinogen's hepatic synthesis, which illustrates its coagulopathic adverse effects.^10^

Developing natural drugs from medicinal plants to hepatic function stimulation, enabling organ protection or hepatic cells regeneration in liver injuries, might be more effective alternative treatments with low toxicity than chemical medications.^11^, ^12^ However, the existence of biological barriers in the body, consisting of drug‐metabolizing enzymes and efflux transporters, could negatively affect the efficiency and pharmacokinetic characteristics of the plant and prevent them from reaching their intended sites of action.^13^ Thus, drug delivery systems (DDs) could be utilized to address these problems. There are various kinds of DDs, such as nanoniosomes. Niosome is a molecular cluster formed by non‐ionic surfactants' self‐association in an aqueous phase. They can load both hydrophilic and lipophilic drugs. As a nanocarrier, they possess several advantages. For example, they are biodegradable, biocompatible, nonimmunogenic and stable in structure. It was proven that they could increase drug bioavailability, absorption and metabolic stability, prolong their circulation in the blood and eventually cause sustained release at the administration site.^14^

*Myristica fragrans* (MF) is an evergreen tropical plant with several pharmacological properties such as anti‐inflammatory, antioxidant, antibacterial, anti‐obesity, antidiabetic, anticancer and hepatoprotective effects. It comprises several phytochemicals like flavonoids, lignans, neolignans, saponins, fatty acids, alkanes, phenylpropanoids, fatty acid esters and steroids.^15^, ^16^ It also has different phenolic compounds showing antioxidative, anxiolytic and anti‐inflammatory activities.^17^ Some experimental studies illustrated the hepatoprotective role of MF against drug‐induced liver injury. For instance, Morita et al. investigated the effect of one of the main nutmeg's important essential oils (myristicin) on LPS/D‐GalN‐induced hepatotoxicity. It showed its prominent hepatoprotective activity through high reduction of DNA fragmentation and TNF‐α's serum concentrations resulting from the drug utilization in mice.^18^ In addition, in another study by Sohn et al., after pretreatment with macelignan, isolating from MF, cisplatin‐induced extracellular signal‐regulated kinase1/2 (ERK1/2) and phosphorylation of c‐Jun N‐terminal kinase1/2 (JNK1/2) was abolished. It indicates the hepatoprotective features of macelignan.^19^ To the best of our knowledge, this is the first study investigating the hepatoprotective role of niosome‐loaded MF phenolic compound on LA‐induced hepatotoxicity in Balb/c mice.

## 2 MATERIALS AND METHODS

### 2.1 Fractionation of MF phenolic compounds

**FIGURE 1:**
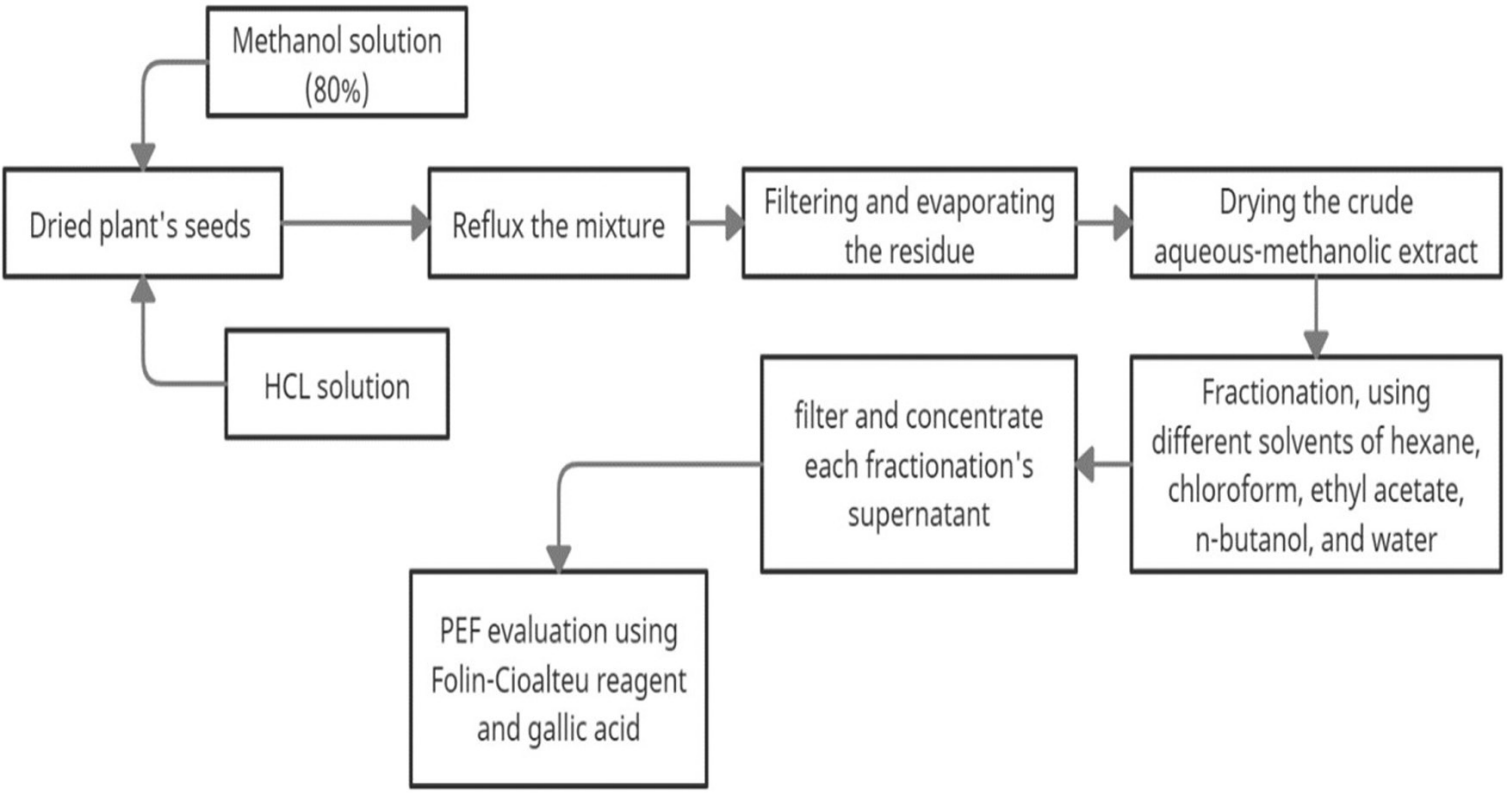
Nutmeg's phenolic compounds fractionation procedure

Initially, a 900 ml aqueous methanol solution of 80% (v/v) and 100 ml of 6 M HCl solution was mixed with 100 grams of dried seeds of MF. We refluxed the mixture for 2 h, then filtered the filtrate and evaporated the residue at 60°C using an evaporator (Buchi, Flawil, Switzerland). After drying the crude aqueous‐methanolic extract, separating funnel and different solvents such as hexane, chloroform, ethyl acetate, n‐butanol and water were used to fractionate it as described by Taghipoor Shamansoor et al^21^ A vacuumed rotary evaporator was used to filter and concentrate the fractionated supernatant after each fractionation step (Figure 1). A phenolic‐enriched fraction (PEF) is the fraction that contains the highest content of phenols. PEF was evaluated quantitatively for total phenolic content using the Folin–Ciocalteu reagent and gallic acid as a standard. We measured the absorbance at 765 nm.

### 2.2 Nanoniosome‐loaded MF phenolic compounds

The NLMP preparation was accomplished based on the thin layer hydration method, which was done in a study conducted by Ghafelebashi et al^21^ Surface‐active agents (1:1 M ratio) and cholesterol (2000 μM) were dissolved in 10 ml chloroform. After that, the MF phenolic extract was added to the solution. A rotatory evaporator with 120 rpm at 60°C for 1 h removed the organic solvent. Then, to gain the niosomal formulation, 10 ml PBS (pH = 7.4) at 120 rpm at 60°C for 1 h was used to hydrate the remaining dried thin films. In the end, 5 min sonication was done to get niosomes with uniform size distribution. The samples were refrigerated (4°C) for subsequent experiments.

### 2.3 The NLMP characterization

Physical parameters of particles like polydispersity index (PDI) and Z‐average were measured by the dynamic light scattering (DLS) method. In addition, the stability of NLMP was specified. Finally, a field emission scanning electron microscope (FESEM) was used to verify the sizes of NLMP particles. Zetasizer Nano ZS‐90 (Malvem, UK) was utilized to measure the NLMP Z‐average, PDI and zeta potential parameters.

### 2.4 Phenolic profiling of nanoniosomes

To identify the phenolic compounds that exist in the nanoniosomes‐loaded PRF of MF, Reversed‐Phase High‐Performance Liquid Chromatography (RP‐HPLC) was utilized. Many phenolic standards (catechin, vanillic acid, naringin, gallic acid, syringic acid, salicylic acid, caffeic acid, pyrogallol, and cinnamic, ellagic acid, chrysin and ferulic acid) were used during this experiment. Here, we used acetonitrile (solvent B) and deionized water (solvent A). Using 85 per cent of solvent A and 15 per cent of solvent B, the column was eluted and equilibrated before injection. After 60 min, the proportion of solvent B was increased to 85 per cent. Having run the experiment for 5 min, the ratio of solvent B had fallen to 15 per cent. The subsequent analysis was conducted at a flow rate of 1 ml/min with a ratio of 70 min. To analyse the content of phenolic at 280 nm, a column (Intersil ODS‐3 5 μm 4.6 × 150 mm Gl Science Inc) was used.

### 2.5 Mice trial

The twenty‐eight male Balb/c mice with average body weight (BW) of 25–30 g (aged 8 weeks) were randomly separated into three groups with 8 mice in each treatment as replicates. The mice were kept in standard animal cages for 7 days at 23°C ± 1°C and 58% ± 10% humidity with alternating 12‐h light and dark periods to adapt to the laboratory conditions. The treatments were as follows: T1 served with the normal regimen without receiving LA and NLMP particles. Hepatotoxicity was induced by LA injection (0.1 ml/kg mice BW) through intraperitoneal administration in two other groups. Then, T2 received a normal regimen, and T3 was fed with an NLMP‐supplemented regimen throughout the experiments for 30 days. Finally, the animals were anaesthetized using chloroform, and blood was collected through cardiac puncture from heart tissue to get serum to study biochemical parameters. All animal experiments were carried out according to the ethical principles approved by the Islamic Azad University, Mashhad, Iran, by the ethics code of IR.IAU.MSHD.REC.1400.069.

### 2.6 Mice's tissues biopsy sampling and pathological studies

After the mice's sacrifice, sampling was performed from the liver and kidney, weighed and washed clean of blood with ice‐cold saline. The liver samples were stored at −80°C for gene expression and lipid peroxidation studies. The organs were fixed within 10% buffered formalin to determine the histopathological changes in the liver caused by LA‐induced hepatotoxicity. After washing the tissues with distilled water, they were paraffinized, sectioned and stained through the haematoxylin/eosin protocol according to the protocol used earlier by D Cardiff et al.^23^ the examination of cell morphology was conducted via a light microscope (Nikon, Japan) at the magnification of × 100.

### 2.7 Blood parameter evaluation

The blood parameters were measured using a kit supplied by the manufacturer. Among the parameters to be measured are markers of liver damage (AST, ALT and ALP), total protein (TP), albumin (Alb), total bilirubin (Total Bil) and lipid profile (TG, Chol and high‐density lipoprotein [HDL]). Immunoglobulins A, G, and M (IgA, IgG, IgM) and antioxidant markers (GPX, CAT and SOD) are also measured. Auto‐analysers (Hitachi 902 and Japan) were used to set their levels.

### 2.8 Lipid peroxidation assay

As described by Beyrami et al.,^23^ we measured lipid peroxidation. Malondialdehyde (MDA) was used as a lipid peroxidation marker by reacting with Thiobarbituric acid (TBA) and producing a reddish colour detectable at 532 nm. MDA concentration is reflected in the intensity of the colour.

### 2.9 Gene expression analysis

To study how NLMP works against LA‐induced hepatotoxicity, the expression of three different genes (SOD, iNOS and IFN‐γ) were measured in the mice's liver tissues. RNeasy Mini kit (Qiagen, Hilden, Germany) was used to extract RNA from crushed mice's liver tissues. Then, Quantitate Reverse Transcription kit (Qiagen, Hilden, Germany) was utilized to synthesize the cDNA libraries of all groups of mice. Next, the house‐keeping gene (β‐actin) and the sets of primer sequences for the target genes (SOD, iNOS, IFN‐γ) were designed (Table 1). For a comparative real‐time PCR (Roche Diagnostics), Qiagen SYBR Green PCR MasterMix (Germany) was used. The following process was used to amplify the targeted genes: 5 min at 95°C (1X), the 20s at 95°C, then 20s at 55°C for, and 25 s at 72°C (35X). The expression of targeted genes was normalized with β‐actin as a reference gene and then with respective genes in the control group. Table 1 illustrates the characteristics of the primer applied in this study.

**TABLE 1:** Primer sets of targeted genes

Gene	Forward (5′ to 3′)	Reverse (3′ to 5′)
SOD	GTCGGCTTCTCGTCTTGCTC	GCTTTCATCGCCATGCTTCC
iNOS	CACCTTGGAGTTCACCCAGT	ACCACTCGTACTTGGGATGC
β‐Actin	CCTGAACCCTAAGGCCAACC	CAGCTGTGGTGGTGAAGCTG

### 2.10 Statics

One‐way analysis of variance (anova) applying the SPSS GLM procedure (Version 21) was used to accomplish the statical analysis. Duncan's multiple range test was used to compare the means. *p* ≤ 0.05 was considered to show a significant difference between means. Analysis was carried out in triplicate, and results were presented as mean values ± standard deviations.

## 3 RESULTS

### 3.1 MF phenolic compound's fractionation

We obtained various amounts of phenolic content from fractionating MF extracts. According to the fraction analysis, the ethyl acetate fraction has the highest phenolic content with a value of 96.2 ± 3.54 mg GAE/g DW, followed by n‐butanol (78.1 ± 5.92) > water (42.6 ± 4.65) > chloroform (27.4 ± 3.65) > hexane (18.9 ± 3.22) mg GAE/g DW. Since it contained the highest amount of phenolic compounds, the ethyl acetate fraction was designated as a PRF and used in subsequent experiments. These results suggest that the polarity of a solvent can affect the extraction process of phenolic compounds. The phenolic compounds have been reported to be moderately polar as a result of which they are attracted to medium polar solvents such as ethyl acetate.^24^ According to other publications, phenolic content is highest in the ethyl acetate fraction compared to other solvents such as n‐butanol, hexane and chloroform. Similarly, Mariem et al.^25^ and Shamansoori et al.^20^ have reported that ethyl acetate fractions from phenolic compounds of *Retama raetam* and *Rheum Ribes* plants are the most enriched fractions in terms of free radical scavenging activity.

### 3.2 The NLMP Characterization

**FIGURE 2:**
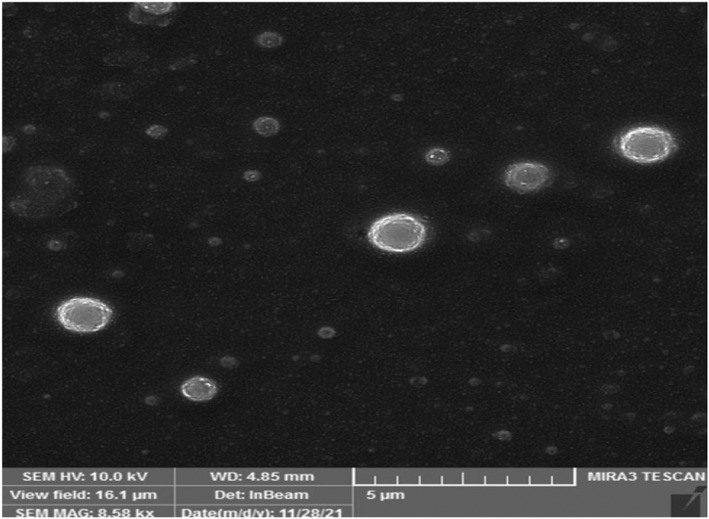
FESEM analysis of NLMP

**FIGURE 3:**
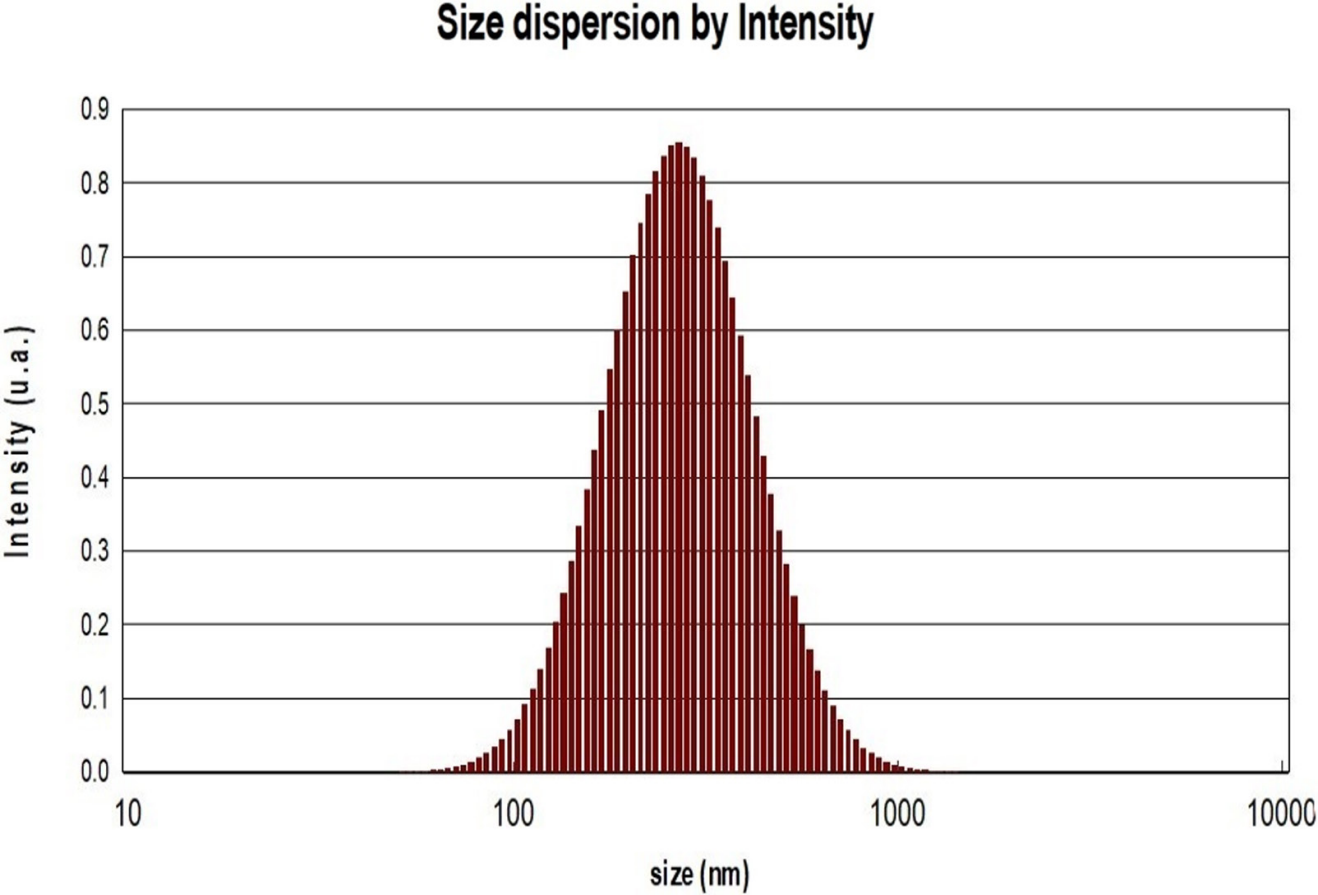
PDI analysis of NLMP

**FIGURE 4:**
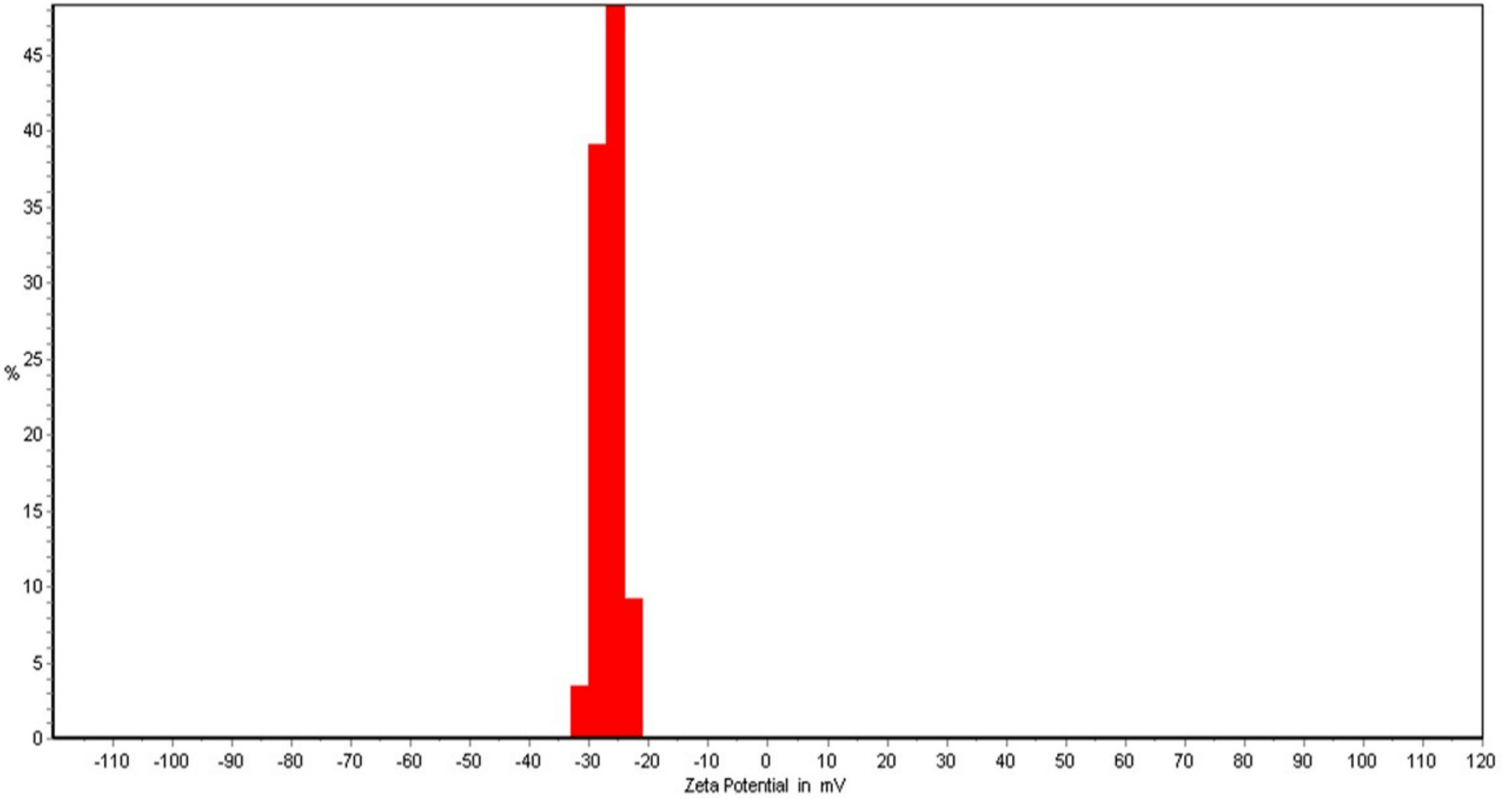
Zeta potential of NLMP

The NLMP physical characteristics comprising the FESEM image, zeta potential and polydispersity index are shown in Figures 2, 3, 4. The result illustrated zeta potential, Z‐average particle size and PDI with values of −26.55 mV, 263 nm and 0.192, respectively. Moderate stability of NLMP colloidal dispersion was revealed from a zeta potential of −26.55. The spherical structure and size of nanoniosomes were proved with the results obtained from the FESEM image and particle size analysis.

### 3.3 Phenolic analysis of PRF‐NLP

The HPLC analysis revealed a variety of natural phytochemicals, including gallic acid, epicatechin, pyrogallol, caffeic acid and ellagic acid, with concentrations ranging from 215 to 716 g/g dried nanoniosomes (Table 2).

**TABLE 2:** Phenolic compounds presented in niosome of *MF*

Phenolic compounds (μg/g DW)				
GA	EP	PY	CA	EA
452.9 ± 3.1	533 ± 6.8	215 ± 5.6	255 ± 3.1	716 ± 7.3

Abbreviations: CA, caffeic acid; EA, ellagic acid; EP, epicatechin; GA, gallic acid; PY, pyrogallol.

### 3.4 The result of the mice trial

During the experimental period, LA‐injected mice gained the least amount of weight by 35.07 mg; however, treating them with NLMP at the concentrations of 2.5 mg/kg led to an increase in their daily weight gain amount significantly (*p* ≤ 0.05) with 48.25 mg (Table 3). the results of changes in body weight corresponded with dietary changes. LA injection curbed the mice appetite; however, NLMP‐supplemented regimen enhanced (*p* ≤ 0.05) it, and thus, their food intake increased to 2.41 grams in response to the NLMP treatment.

**TABLE 3:** Averages of mice body weight changes and feed intake during experiment receiving different treatments

Average	T1	T2	T3	SEM
Average daily weight gain (mg/day)	53.88^a^	35.07^c^	48.25^b^	3.89
Average daily feed intake (g)	3.26^a^	1.67^c^	2.41^b^	0.13

*Note*: T1, control; T2, LA‐injected mice; T3: NLMP‐treated mice. Different letters in the same raw indicated significant difference (*p* < 0.05). The analysis was performed in triplicates.

### 3.5 Mice blood parameters

The AST, ALT and ALP are liver enzymes considered biomarkers, indicating liver function and health. TG, fatty acids and free cholesterols are also contributed to liver damage. Lipid accumulation in hepatocytes induces liver damage and provokes inflammation, fibrosis and cirrhosis.^27^ Although these blood factors were perturbed by LA, NLMP could modulate them by significantly reducing TG and Chol levels, and increasing HDL levels (*p* ≤ 0.05). The importance of total protein (TP), serum albumin (Alb) and bilirubin (Bil) was highlighted as tests that are enable showing liver diseases. After treatment with NLMP, Alb and TP were increased except for total Bil, though their alteration was not meaningful. Antioxidant factors such as GPX, SOD and CAT were also measured in the blood (Table 4). They all have risen significantly (*p* ≤ 0.05) following the treatment with NLMP in a way that GPX and CAT magnitude reached their peak even higher than in control groups (Table 4).

**TABLE 4:** Blood parameter analysis during experiment receiving different treatments

Parameters	T1	T2	T3	SEM
TG (mg/dl)	122b	127a	124b	1.95
Chol (mg/dl)	151c	175a	165b	2.78
HDL (mg/dl)	40b	32c	57a	3.67
TP (g/dl)	5.3a	5.5a	6.2a	1.12
Alb (g/dl)	2.7a	3.4a	3.6a	0.14
AST (U/L)	191b	256a	187c	3.48
ALT (U/L)	107c	168a	127b	4.29
ALP (U/L)	103c	324a	132b	3.76
Total Bil (mg/dl)	0.32c	0.61a	0.58b	0.03
IgA (mg/dl)	7.8a	6.1b	7.9a	0.28
IgG (mg/dl)	7.7a	7.2b	8.1a	0.13
IgM (mg/dl)	6.1a	5.2b	6.4a	0.17
GPX (U/ml)	654b	589c	701a	6.38
SOD (U/ml)	55a	41c	49b	1.79
CAT (U/ml)	28b	17c	35a	3.48

*Note*: T1, control; T2, LA‐injected mice; T3, NLMP‐treated mice. Different letters in the same raw indicated significant difference (*p* < 0.05). The analysis was performed in triplicates.

### 3.6 Lipid peroxidation

**FIGURE 5:**
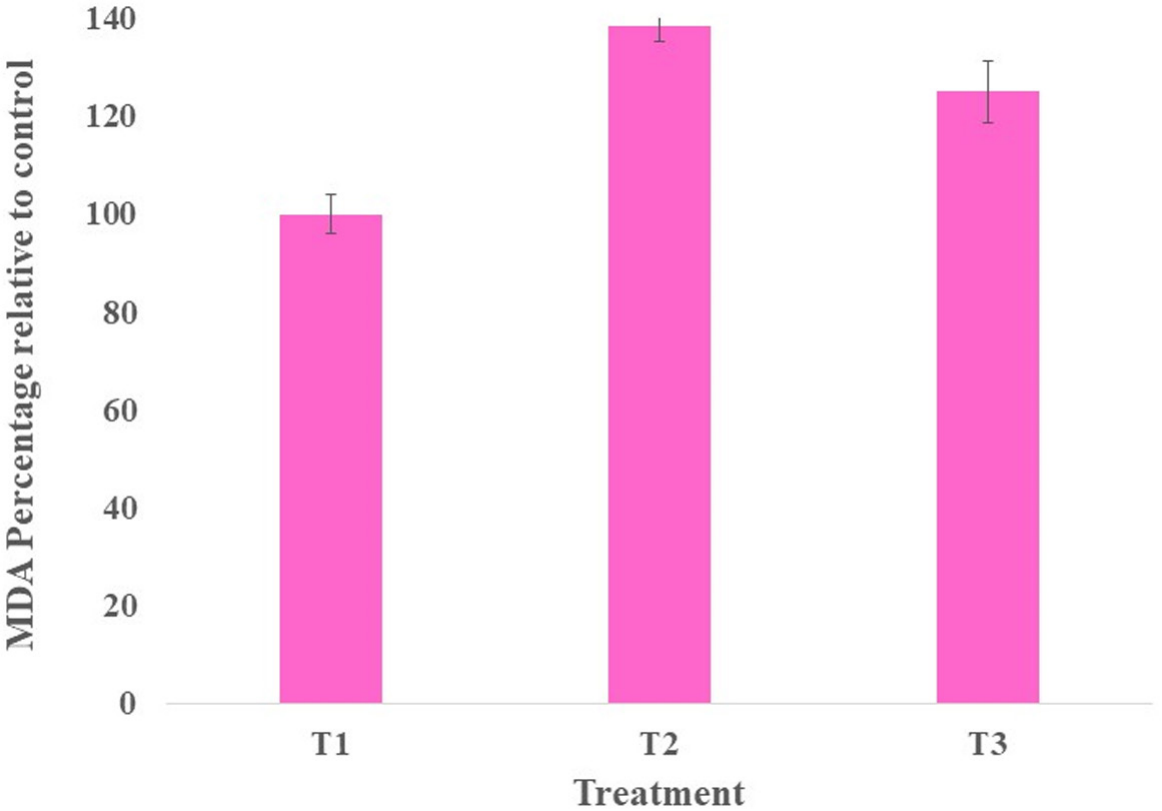
Changes in MDA levels in mice liver received different treatments

The result of lipid peroxidation is shown in Figure 5. The results indicated that the lipid peroxidation increased significantly (*p* ≤ 0.05) in the liver due to the injection of AL. Treatments with NLMP have led to the meaningful diminution of lipid peroxidation value (*p* ≤ 0.05).

### 3.7 The gene expression analysis

Table 5. demonstrates the expression analysis of the main antioxidant, inflammatory and immune‐related genes (SOD, iNOS, IFN‐γ). The supplementation of NLMP in the mice regimen (*p* < 0.05) increased the expression of SOD (antioxidant‐related gene), which was decreased due to the LA exposure. Up‐regulation of iNOS gene expression in the T2 affirmed the existence of inflammation in the mice's liver. However, the treatment of mice with NLMP decreased the expression of the iNOS gene (*p* ≤ 0.05) and should be associated with the diminution of liver inflammation. Changes in the expression of IFN‐γ were also indicated in Table 5. It was meaningfully decreased (*p* < 0.05) after treatment with NLMP.

**TABLE 5:** Changes in the expression of antioxidant‐related genes in mice liver received different treatment

Gene expression (Fold changes)				S.E.M
Genes	T1	T2	T3	
SOD	1.0^c^	‐3.8^a^	−2.1^b^	0.03
iNOS	1.0^c^	+2.9^a^	+1.6^b^	0.06
INF‐gamma	1.0^c^	+3.4^a^	+2.1^b^	0.07

*Note*: T1: Control; T2: LA‐injected mice; T3: NLMP‐treated mice. Means (*n* = 3) with different superscripts within a row are significantly different (*p* < 0.05). Abbreviation: S.E.M: standard error of the means.

### 3.8 Histopathological features of liver and kidney

**FIGURE 6:**
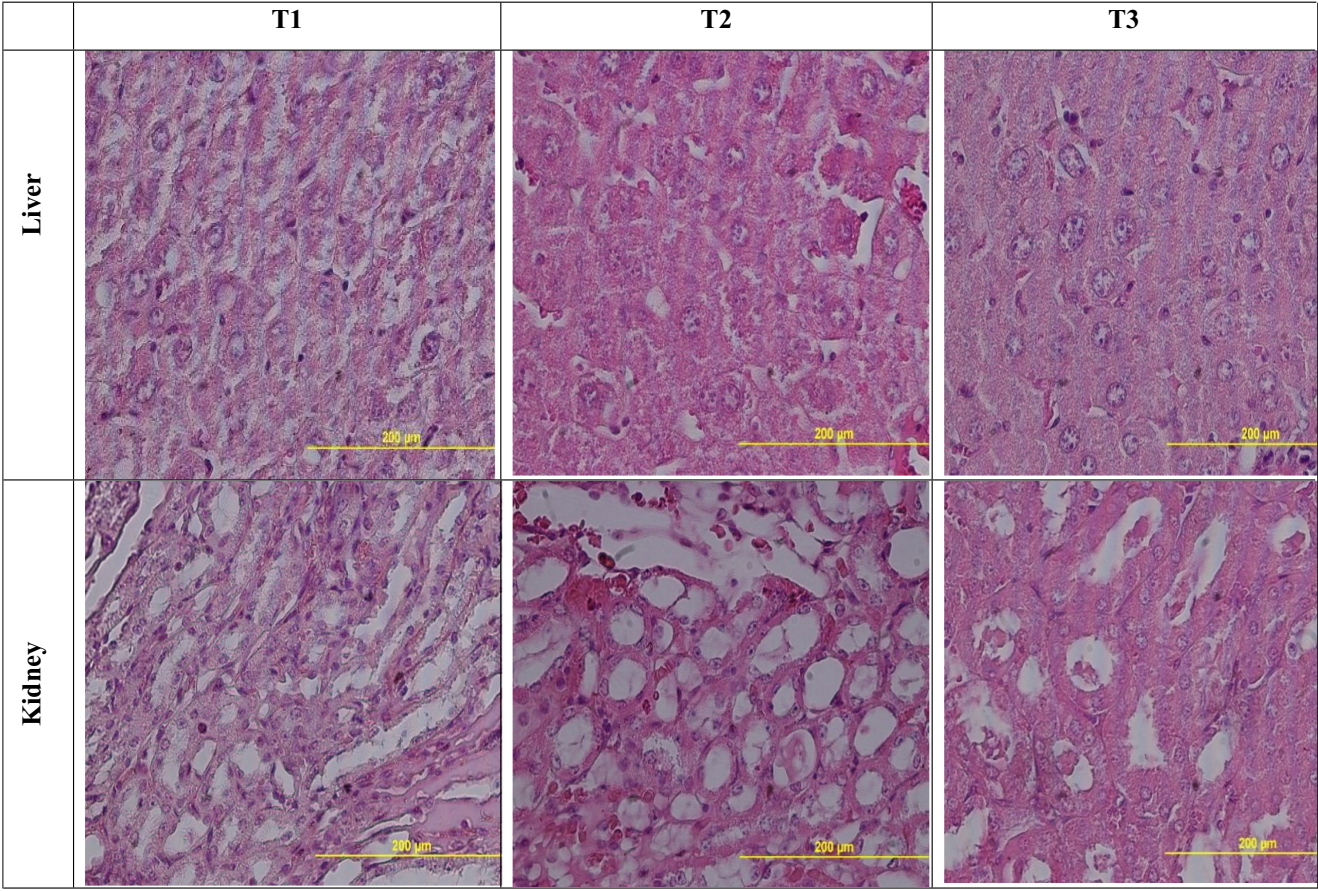
Histopathological changes in the mice liver and kidney received different treatments. T1: control; T2: LA‐injected mice; T3: NLMP‐treated mice

Histopathological assessment of the liver and kidney damage resulting from LA was done to evaluate the protective effects of NLMP treatment. Figure 6 shows that the liver tissue was intact, the hepatic lobules were clear, and the hepatocytes were arranged regularly. Loss of basic cellular architecture of liver cells was detected in the LA‐injected group, and liver cells were swollen and infiltrated with inflammatory cells. As opposed to this, NLMP significantly reduced the severity of liver histopathological changes and liver cells through decreasing swollen, infiltrated and inflammatory cells. According to Figure 6, the control group did not have any histopathological abnormalities in the kidney. A variety of adverse histopathological alterations happened after LA injection. NLMP treatment could decrease those harmful effects including oedema, inflammatory cell infiltration, enlarged glomeruli, narrowed renal tubules, swelling, degeneration, and epithelial cells falling and preserved the renal architecture (Figure 6).

## 4 DISCUSSION

Many drugs, such as LA as an anticancer agent, are identified to cause drug‐induced hepatotoxicity, which is an important concern in drug development as it can lead to the loss of hepatic functionality and acute liver failure.^27^ Synthetic hepatoprotective remedies are not therapeutically promising since they can even lead to liver toxicity. However, medicinal plants and their bioactives, which have been utilized to treat liver diseases for a long time, are considered to be relatively safe.^28^ MF is a medicinal plant, and its hepatoprotective features have been proved in some researches. A Dkhil et al. reported that MF Kernels inhibit Paracetamol‐induced liver toxicity through induction of anti‐apoptotic genes and Nrf2/HO‐1 pathway.^29^ In another survey by Yang et al., nutmeg could indicate its hepatoprotective activity against thioacetamide‐induced acute liver injury in mice by increasing serum transaminases, decreasing hepatic oxidative stress, and hepatic inflammation and these protective features are dependent on peroxisome proliferator‐activated receptor alpha PPARα.^30^

Nanotechnology has been a promising addition to natural medicine. New methods of delivering plant extracts and poorly soluble phytochemicals have improved pharmacokinetics and clinical outcomes.^32^ Encapsulation of drugs in niosomes has been utilized for different purposes. In the present work, we entrapped the MF phenolic compound into nanoniosomes.

The particle size and zeta potential are two important parameters that characterize colloidal drug delivery systems, and their effects on the stability of the carriers are a well‐established relationship.^27^, ^28^ In order to prevent the aggregation of colloidal systems, it is necessary to provide some barrier between the particles, such as steric or static barriers or introduce a charge on the surface of the vesicles. Zeta potential is an indicator of the size of this barrier. If all of the particles have large enough zeta potential, the particles may repel each other strong enough not to tend to come together.^32^ PDI, which is reported at 0.192, is also an indicator of the sample's heterogeneity based on size. It ranges from 0.0 to 1 for a sample with multiple particle size populations. Polymer‐based nanoparticle materials are usually considered acceptable, with values of 0.2 and below.^33^, ^34^

In this study, LA influenced the mice's food intake and body weight. Reduction in weight and food intake could be linked to the effect of LA on appetite.^35^ However, an increase in the mice's food intake and weight after treatment with NLP is due to the appetite‐enhancing feature of its phenylpropanoids such as elemicin, myristicin and methyl eugenol.^36^ In addition, the LA caused toxicity in the liver function as it elevated the liver enzymes and bilirubin. In a study by Lee et al.,^37^ it is reported that LA led to liver malfunction through elevation in ALT and AST serum levels. In agreement with our study, research done by Zhao et al.^38^ indicated that utilization of the MF extract in mice diet could alleviate hepatocyte steatosis by decreasing the blood levels of ALT, AST and ALP. In the present study, LA adversely affected TG, cholesterol and HDL serum levels. Various studies have reported the adverse effect of the LA on lipid profile through increasing the HDL, cholesterol and especially TG. There is a hypothesis that the ASP increases the synthesis of endogenous very low‐density lipoprotein (VLDL) and exogenous chylomicrons by causing a decrease in lipoprotein lipase activity. A disturbance in lipoprotein metabolism has also been associated with ASP.^39^, ^40^ Our results showed that the lipid profile was decreased after treatment with NLMP. It has been reported that therapy with alcoholic nutmeg's extract can inhibit the expression of genes related to lipid synthesis, such as fatty acid synthase (FASN) and sterol regulatory element‐binding protein 1c (SREBP‐1c), resulting in a lower lipid content within the cells. FASN is also contribute to the liver triglyceride metabolism so MF could decrease the TG levels, which have been increased after LA injection.^38^, ^41^

In the current study, TP and Alb serum levels were increased after treatment with LA. However, it was not significant. In contrast, Merlen et al. illustrated that TP and Alb levels in plasma decreased after patients' treatment with LA.^42^ Due to albumin's oncotic properties, treating liver cirrhosis with albumin has been widely used to expand plasma volume and increase practical circulation volume, thereby alleviating cardiocirculatory changes caused by portal hypertension.^43^ TP and Alb serum levels were increased after NLMP therapy, but it was not meaningful. Hartanto et al.’s results were the same as ours as the TP and Alb serum levels were not significantly changed after treatment of Korean native chicken with dietary nutmeg oil.^44^ In addition, after the LA injection, serum levels of immunoglobulins (IgA, IgG, IgM) increased to enhance the body's defence like the situation which was reported in the study done by Zajaçzkowski et al. and patients who utilized LA had increased levels of serum immunoglobulins.^45^ Treatment with NLMP led to an incredible increase in serum levels of immunoglobulins and significantly enhanced the body's immunity system.

LA injection decreased antioxidant activity by diminishing GPX, SOD and CAT serum levels. However, in another study by El‐Gendy et al., extracellular LA produced by fusarium exquisite AHMF4 showed effective dose‐dependent antioxidant activity through the DPPH radical scavenging test, which has been related to its fungal source, but they have not measured serum antioxidants levels.^46^ After treatment with NLMP, serum levels of antioxidants were significantly increased beyond their normal rates, which could reveal great MF antioxidant activity. Pashapoor et al. also reported that MF Administration for diabetic rats lessened oxidative stress and ameliorated the antioxidant activities in pancreatic tissue through increasing GPX, SOD and CAT levels.^47^

It has been proven that reactive oxygen species, which indicate oxidative stress, can induce lipid peroxidation, which further demonstrates increased MDA levels. The peroxidative breakdown of membrane lipids is related to subcellular and tissue aspects of liver injury. LA injection increased lipid peroxidation in the mice liver, like a study by Kaya et al. in mice pancreas.^49^, ^50^ However, after NLMP treatment, MDA levels in mice livers were decreased significantly. Our findings support the previous findings that stated the presence of tannins and flavonoids in the MF, which act as electron donors to scavenge free reactive oxygen species.^51^

LA injection could change the expression of many liver genes, including SOD, iNOS and IFN‐γ. SOD was decreased after LA injection, and this result is the opposite of a previous study by Suresh et al. that showed that LA increased the gene expression of SOD2 in zebrafish.^6^ NLMP treatment ameliorated the SOD gene expression, which its regulating is key to maintaining a balance in ROS concentrations.^52^ This result is in line with previous investigations that confirmed the antioxidant properties of acetone, ethanol, methanol, butanol and water extracts of the seeds of MF.^16^

One of the direct consequences of inflammation is the expression of inducible nitric oxide synthase (iNOS). Early studies suggested that the high‐level NO‐synthesis was potentially toxic during chronic inflammation. Therefore, it is evident that LA caused inflammation in the mice's liver as it increased the expression of mice's liver iNOS.^52^ In this study, NLMP has decreased iNOS gene expression of the liver compared with those injected with LA. In line with our results, Cao et al. reported that myrisfrageals A and B and four known compounds, 30 ‐methoxylicarin B, licarin B, dehydrodiisoeugenol and isodihydrocainatidin from nutmeg, indicated inhibition upon NO production in LPS‐activated murine monocyte–macrophage RAW 264.7 in a concentration‐dependent manner. In addition, myrisfrageals A and B and dehydrodiisoeugenol significantly inhibited the expression of iNOS mRNA.^53^ In the present study, LA injection increased the immune‐related gene expression of IFN‐γ. IFN‐γ has been shown to increase the response of hepatic progenitor cell to injury by stimulating hepatic inflammation and exacerbating liver damage.^54^ However, NLMP could decrease the expression of IFN‐γ, which is in agreement with the results of the study by Long et al^55^ They have pointed out that TNF‐α and IFN‐γ were markedly and dose‐dependently reduced after treatment with macelignan in rats with ischaemia–reperfusion injury.^55^

Results of histopathological examination showed the protective effects of NLMP treatment with relieving liver and kidney histopathological changes. We identified disintegrated hepatocytes, pyknotic nucleus, congested central vein, marked vacuolated hepatocytes in the liver, infiltration of inflammatory cells, glomeruli enlargement, degeneration and epithelial cells falling in the kidney after LA injection. After treating the mice with NLMP, we found that the disintegrated hepatocytes, pyknotic nuclei and vacuolated hepatocytes were reduced in the liver and degeneration in the kidney. A more detailed understanding of the molecular mechanisms involved in the hepatoprotective potential of NLMP would require the exploration of the protein expression of different apoptotic, inflammatory and fibrotic markers via Western blotting or RT‐PCR for further strengthening the findings.^57^

## 5 CONCLUSION

In conclusion, NLMP were found to possess protective effects against LA‐induced liver injury in mice. The NLMP may exert its protective effects through its ability to lower liver enzymes (ALT, AST, ALP) and inflammation, increase antioxidant activity and improve histopathological alterations in the mice's liver and kidney. These findings imply that NLMP may prove to be a worthwhile therapeutic candidate for preventing and treating liver disease caused by anticancer drug consumption.

## AUTHOR CONTRIBUTIONS

**Mahsa Rastegar Moghaddam Poorbagher:** Conceptualization (equal); formal analysis (equal); methodology (equal); software (equal); writing – original draft (equal). **Ehsan Karimi:** Conceptualization (equal); project administration (equal); software (equal); supervision (equal); writing – original draft (equal); writing – review and editing (equal). **Ehsan Oskoueian:** Formal analysis (equal); project administration (equal); supervision (equal); writing – original draft (equal); writing – review and editing (equal).

## CONFLICT OF INTEREST

The authors have no conflicts of interest to declare.

## Data Availability

The datasets applied during the current study are available on reasonable request.
